# Supplementary material for: The Gene ANTHER DEHISCENCE REPRESSOR (ADR) Controls Male Fertility by Suppressing the ROS Accumulation and Anther Cell Wall Thickening in Arabidopsis
Source: Sci Rep. 2019 Mar 25;9:5112. doi: 10.1038/s41598-019-41382-z (PMC6434047; doi:10.1038/s41598-019-41382-z)
Supplement: Supplementary file 1 — Supplementary Figs and Table [file 41598_2019_41382_MOESM1_ESM.pdf]

The Gene *ANTHER DEHISCENCE REPRESSOR* (*ADR*) Controls Male Fertility by Suppressing the ROS Accumulation and Anther Cell Wall Thickening in *Arabidopsis*

Shu-Yu Dai[1], Wei-Han Hsu[1] and Chang-Hsien Yang[1,2*]

[1]Institute of Biotechnology, [2]Advanced Plant Biotechnology Center,
National Chung Hsing University,
Taichung, Taiwan 40227 ROC

* To whom correspondence should be addressed
E-mail: chyang@dragon.nchu.edu.tw

Abstract

Male sterility in plants is caused by various stimuli such as hormone changes, stress, cytoplasmic alterations and nuclear gene mutations. The gene *ANTHER DEHISCENCE REPRESSOR* (*ADR*), which is involved in regulating male sterility in *Arabidopsis*, was functionally analyzed in this study. In ADR::*GUS* flowers, strong GUS activity was detected in the anthers of young flower buds but was low in mature flowers. ADR+GFP fusion proteins, which can be modified by N-myristoylation, were targeted to peroxisomes. Ectopic expression of *ADR* in transgenic Arabidopsis plants resulted in male sterility due to anther indehiscence. The defect in anther dehiscence in 35S::*ADR* flowers is due to the reduction of ROS accumulation, alteration of the secondary thickening in the anther endothecium and suppression of the expression of *NST1* and *NST2*, which are required for anther dehiscence through regulation of secondary wall thickening in anther endothecial cells. This defect could be rescued by external application of hydrogen peroxide (H_2O_2). These results demonstrated that ADR must be N-myristoylated and targeted to the peroxisome during the early stages of flower development to negatively regulate anther dehiscence by suppressing ROS accumulation and *NST1*/*NST2* expression.

Introduction

Anther dehiscence is an important process in mature stamens. In this process, lignin accumulation in the endothecium of anthers enables secondary wall thickening. Subsequently, septum and stomium lysis completes dehiscence[1-6].

The expansion of the endothecium provides an internal directed force for anther dehiscence, causing breakdown of the stomium. Then, desiccation of the epidermis causes differential shrinkage of thickened and unthickened parts of the cell wall, resulting in an outwardly bending force that leads to the retraction of the anther wall and the complete opening of the stomium[7-9]. Lignin is the major compound involved in secondary wall thickening in anthers, and its polymerization is dependent on hydrogen peroxide (H_2O_2) levels[10] which is the major ROS form in plant cells and the substrate for peroxidase in catalyzing lignin polymerization[11-14]. The final polymerization steps of lignin biosynthesis occur after the activation of monolignols to free radicals, which is mediated by peroxidase + H_2O_2 and/or oxidase (laccase) + O_2, followed by non-enzymatic coupling of monolignol radicals to form the polymer lignin[11-15]. Interestingly, H_2O_2 also plays an important role as a signal in activating transcription of lignin biosynthesis enzyme such as peroxidases[16]. It has been reported that catalases (CATs) are the other major H_2O_2-scavenging enzymes which are located in peroxisomes[17]. In a previous study, the NAC transcription factors *NAC SECONDARY WALL THICKENING PROMOTING FACTOR1* (*NST1*) and *NST2* were reported to regulate lignin synthesis of anther secondary walls[18]. Double mutations in *NST1* and *NST2* caused sterility due to anther indehiscence.

Although it is well established that the H_2O_2 level in the anther endothecium is strongly correlated with the lignin polymerization and anther dehiscence, the mechanisms regulate H_2O_2 level in the anther still remain to be investigated. In cells, the H_2O_2 can be accumulated in organelles such as peroxisomes, which are single membrane-bound organelles with diverse metabolic functions[19,20]. In

association with ROS production, peroxisome targeted PAO (polyamine oxidase) has been reported to regulate pollen tube elongation[21]. A mutation in the peroxisomal membrane protein DAYU impairs pollen maturation and germination[22]. However, the association between anther dehiscence and peroxisomes remains unclear. Therefore, it is interesting to explore the mechanisms or factors that regulate or affect H_2O_2 accumulation in peroxisomes.

To explore the association between H_2O_2-regulated anther dehiscence and peroxisomes, a novel *ANTHER DEHISCENCE REPRESSOR* (*ADR*) gene (At4g13540), which is potentially N-myristoylated and targeted to peroxisomes, was cloned from *Arabidopsis* and analyzed. The ADR protein contains a predicted conserved recognition site (GGSTSKD) for N-myristoylase[23] at the N-terminus of the protein. It has been reported that the N-terminal octapeptide of ADR can be myristoylated by AtNMTs (N-myristoyltransferase)[23]. ADR also contains a binding site for a peroxisomal targeting signal (PTS) in the middle of the protein, which indicates that ADR is likely targeted to the peroxisomes. The PTS binding site is critical for protein targeting and binding to peroxisomal matrix proteins (Pex5 and Pex7) or peroxisomal membrane proteins (Pex19) and thus allows the peroxisome entry or peroxisomal membrane association[24]. N-myristoylation involves the addition of the saturated C:14 fatty acid myristate to the N-terminus of proteins and affects the membrane binding properties of proteins[23,25]. A mutation in the myristoylation domain did not interfere with peroxisomal targeting but disrupted the membrane association of proteins because it prevented the addition of the myristoyl group that is also essential for membrane association. Based on these observations, proteins lacking a myristoyl group can still bind to Pex through the PTS binding site and target to the peroxisome, but they cannot stably associate with the peroxisomal membrane. In this study, we demonstrated that ADR proteins are likely modified by N-myristoylation and targeted to peroxisomes. We showed that ectopically expressing *ADR* causes male sterility of the flowers due to anther indehiscence.

We also found that *ADR* functions to reduce ROS accumulation and suppresses the expression of *NST1* and *NST2*. Thus, we propose a model in which ADR is myristoylated and negatively regulates anther dehiscence by promoting ROS scavenging in the peroxisome, which affect lignin polymerization and stomium rupture in Arabidopsis.

Results

Isolation of *ADR* cDNA from Arabidopsis

ADR contains 2 exons and 1 intron and encodes a protein of 210 amino acids (Fig. S1). A predicted conserved recognition site (GGSTSKD) for N-myristoylase (23. Pierre et al. 2007) and several basic residues reported to stabilize membrane binding[26] were identified at the N-terminus of the ADR proteins (Fig. S1). A binding site for a peroxisomal targeting signal (PTS) predicted using the PTSs Target Signal Predictor (http://216.92.14.62/Target_signal.php) was also found in the middle of the protein (Fig. S1). In contrast, no MTS (mitochondria targeting sequence) was identified in ADR using the prediction tool MitoFates (http://mitf.cbrc.jp/MitoFates/cgi-bin/top.cgi). The ADR protein showed 68% identity and 78% similarity to the most closely related ADR-like protein, At3g23930 (Fig. S1). In their N-myristoylase sites, 95% of the amino acids are identical (Fig. S1).

RT-PCR analysis of *ADR* transcripts and detection of *ADR* expression by analyzing ADR::*GUS* transgenic Arabidopsis plants

Reverse transcription PCR (RT-PCR) was performed to determine the relative transcript abundance of *ADR* at different developmental stages and in various

organs of *Arabidopsis*. *ADR* expression was not detected in early seedling development (**Fig. 1A**). The transcript level of *ADR* was strongly detected in flowers and weakly detected in the roots, stem and siliques, but *ADR* transcripts were absent in the leaves of mature plants (**Fig. 1A**). When the expression of *ADR* in flowers at different developmental stages was further analyzed, significantly higher expression of *ADR* was observed in early development stages (stages 8-11) than in late flower development stages (after stage 12; **Fig. 1B**).

To further analyze the expression pattern of the *ADR* gene in flowers, a construct (ADR::*GUS*) was generated and transformed into *Arabidopsis*; twelve independent ADR::*GUS* plants were obtained. GUS activity in the ADR::*GUS* flowers was strongly detected in sepals but was relatively weakly detected in petals and carpels during early and late flower development (**Fig. 1C, E**). In the stamen, GUS activity was strongly detected in anthers during early flower development stages (before stage 10; **Fig. 1C, D**), but its expression was almost undetectable in anthers during late developmental stages (**Fig. 1E, F**).

ADR needs to targeted to peroxisomes to perform its function

It has been shown that the N-terminus of ADR can be myristoylated by an in vitro myristoylation assay after the first eight residues of the N-terminal peptide sequence recognized by *N*-myristoyltransferase[23]. Because N-myristoylation is known to affect the membrane-binding properties of proteins[25], an Agrobacterium infiltration-mediated transient expression assay was performed to validate the membrane association properties of ADR. The cDNA of *ADR* fused with *GFP* driven by the cauliflower mosaic virus (CaMV) 35S promoter (*ADR+GFP*) were transformed into *Agrobacterium* and infiltrated into the leaf epidermis of *Nicotiana benthamiana*. The results showed that ADR+GFP fusion proteins mainly accumulated in organelle-like structures (**Fig. 2A, B**) which may be the peroxisomes since ADR contains a binding site for a peroxisomal targeting

signal (PTS) as described above (Fig. S1). To examine that the organelle-like structures were peroxisomes, a construct of *CATALASE 3* (*CAT3*) fused to *mORANGE2* (*CAT3+mORG2*) was co-transformed with *ADR+GFP* into *Agrobacterium* and infiltrated into the leaf epidermis of *N. benthamiana*. It has been shown that CAT3 could localize to the peroxisomes[27] (**Fig. 2C, D**). Confocal images showed that ADR+GFP co-localized with CAT3+mORG2 (**Fig. 2E, F**), indicating that myristoylated ADR is likely associated with peroxisomes. Both ADR+GFP and CAT3+mORG2 were also detected in the nucleus (**Fig. 2A, C, E**). Because it has been reported that the maximum protein size that can diffuse freely through the nuclear pore is larger than 60 kDa[28], the diffusion of these two proteins (both are smaller than 60kD) into the nucleus may be due to the over-expression of high amounts of the proteins in these cells.

It has been reported that some proteins containing MTS (mitochondria targeting sequence) are targeted to the mitochondria after N-myristoylation[29]. To further confirm the localization of ADR is not in other organelles such as mitochondria, a construct of the mitochondria marker *MT* fused to *mCherry* (*MT+RK*) was co-transformed with *ADR+GFP* into *Agrobacterium* and infiltrated into the leaf epidermis of *N. benthamiana*. Confocal images showed that ADR+GFP (**Fig. 3A, B, E, F**) did not co-localize with MT+RK (**Fig. 3C, D, E, F**), indicating that myristoylated ADR is not associated with mitochondria.

Ectopic expression of *ADR* causes plant sterility due to anther indehiscence

To investigate the function of the *ADR* gene, the cDNA of the *ADR* gene driven by the cauliflower mosaic virus (CaMV) 35S promoter (35S::*ADR*) was transformed into *Arabidopsis*. A total of sixteen 35S::*ADR* transgenic *Arabidopsis* plants showing similar abnormal phenotypes were obtained. When the inflorescence was examined, a sterile flower phenotype with the siliques failing to elongate during late development was observed in these 35S::*ADR*

transgenic plants (**Fig. 4A, B, C**). The severity of the sterile phenotype in the 35S::*ADR* plants was correlated with the *ADR* expression level (**Fig. 4C, D**).

When the 35S::*ADR* flowers (**Fig. 4E**) were examined, they were similar to wild-type flowers (**Fig. 4F**), opening normally and producing normal sepals, petals and carpels with fully developed stigmatic papillae. The anthers of the 35S::*ADR* flowers were indehiscent at all stages of flower development (**Fig. 4E, G**). In contrast, wild-type anthers were dehiscent, and pollen released after stage 12 of flower development (**Fig. 4F, H**). The 35S::*ADR* flowers with severe phenotypes were sterile and unable to set seed due to the indehiscence of anthers throughout flower development (**Fig. 4C**). To further examine pollen viability, Alexander's stain, which can distinguish viable pollen from nonviable pollen[30], was applied. Normal viability (dark blue staining), similar to that of wild-type pollen (**Fig. 5A**), was observed in the 35S::*ADR* pollen (**Fig. 5B**). When further examined by SEM, the pollen grains from wild-type anthers and 35S::*ADR* indehiscent anthers exhibited a similar egg shape, 30 x 5 micrometers in size (**Fig. 5C, D**). These results indicate that the pollen produced in the 35S::*ADR* flowers was still functional.

To examine pistil function, 35S::*ADF* stigmas were manually pollinated with pollen from wild-type flowers. Elongated and fully developed siliques (**Fig. 5E, F**) with normal seed development (**Fig. 5G**) were observed. This result confirms that the sterility of the 35S::*ADR* flowers is due to the indehiscence of the anther.

To further determine the role of *ADR* in regulating anther dehiscence, an *ADR* loss-of-function T-DNA insertion line, SALK_072305 (containing an insertion in the 5' promoter region) (Fig. S2A), was analyzed. The result indicated that these *ADR* mutants were phenotypically indistinguishable from wild-type plants in both vegetative and reproductive development (Fig. S2B). Furthermore, the anthers in these *ADR* mutants were dehiscent normally (Fig. S2C, D) similar to that in wild-type plants. This finding indicates a possible functional redundancy between *ADR* and other unknown genes. Further analysis

indicated that the expression of *ADR* in these T-DNA insertion mutants was significantly reduced (Fig. S2E).

Ectopic expression of *ADR-Gly* resulted in normal fertility

To confirm the myristoylated ADR was associated with the sterile phenotype observed in 35S::*ADR* plants, a mutated ADR that lacks the N-terminal myristoylation site Gly (35S::*ADR-Gly*) was transformed into *Arabidopsis*, and the phenotype was analyzed. Unlike 35S::*ADR* plants, 35S::*ADR-Gly* plants showed normal fertility, similar to wild-type (Fig. S3A, B). Elongated and fully developed siliques that clearly differed from those of 35S::*ADR* flowers (Fig. S3A, B, D) were observed in 35S::*ADR-Gly* inflorescence (Fig. S3B, C). Upon further examination, the anthers of 35S::*ADR-Gly* plants, which were normal dehiscent anthers that were distinct from those of 35S::*ADR* plants (Fig. S3E, F), were observed (Fig. S3G, H). These results indicated that the sterile phenotype of 35S::*ADR* plants is correlated with the myristoylation.

The secondary wall thickening was affected in 35S::*ADR* anthers

In the process of anther dehiscence, lignification enables the secondary wall thickening in endothecial cells of the anther[15], it is possible that the lignification of the endothecial cells of the 35S::*ADR* anthers is affected.

To further analyze the cellular basis for anther dehiscence and to examine the formation of secondary wall thickness, lignin staining with auramine O was performed on the endothecium of developing anthers in both 35S::*ADR* and wild-type plants. The result indicated that secondary thickening occurs in the endothecium before anther dehiscence, and the surrounding cell layers of the anther did not undergo secondary thickening during wild-type anther development (**Fig. 6A, B**) [6,7]. In contrast, no secondary thickening or lignification

was observed in the anther endothecium of 35S::*ADR* plants (**Fig. 6C, D**). This result indicates that the developmental processes of anther dehiscence in 35S::*ADR* plants is interrupted by the failure of lignification as well as secondary wall thickening in endothecial cells of the anthers.

The expression of *NST1/2* that participate in lignin accumulation of anther secondary walls was down-regulated in 35S::*ADR* transgenic Arabidopsis

It has been previously reported that two *NAC*-like genes, *NAC SECONDARY WALL THICKENING PROMOTING FACTOR 1* (*NST1*) and *NST2*, are involved in the regulation of male sterility in plants. Double mutants of *nst1/nst2* cause male sterility and have a similar anther-indehiscent phenotype[18] as that of 35S::*ADR* plants. It has been reported that *NST1* and *NST2* regulate secondary wall thickening in various tissues, and anther dehiscence in the *nst1/nst2* double mutants is due to the loss of secondary wall thickening in the anther endothecium[18].

It is also interesting to explore whether the ectopic expression of *ADR* affects the expression of *NST1/2* genes in transgenic plants and causes the alteration of anther dehiscence. Thus, the expression of these two genes was analyzed from the flowers of 35S::*ADR* transgenic plants by real-time quantitative RT-PCR analysis. The result indicated that the transcript levels of the *NST1* and *NST2* were significantly down-regulated in the flowers of 35S::*ADR* plants (**Fig. 6E**). This result indicates that altered anther dehiscence in 35S::*ADR* plants is correlated with the altered expression of *NST1/2* that participate in the regulation of secondary wall thickening in anthers.

ROS accumulation was lower in 35S::*ADR* anthers than in wild-type anthers

It has been known that the lignification of the secondary wall thickening in

endothecial cells of the anther depends on ROS level[15]. The peroxisome has been thought to be the major location of reactive oxygen species (ROS) scavenging in plant cells[31]. Since the lignification of the endothecial cells of the 35S::*ADR* anthers is affected, it is possible that the H_2O_2 accumulation in 35S::*ADR* anthers is altered. To confirm whether ADR associates with the peroxisome and further affects ROS accumulation, endogenous H_2O_2, which is the major ROS form in plant cells, in 35S::*ADR* and wild-type anthers at different developmental stages was detected directly by 3',3'-diaminobenzidine (DAB), which is oxidized by H_2O_2, generating a dark brown precipitate[32-34]. The results indicated that a significant reduction of H_2O_2 accumulation was observed in 35S::*ADR* anthers in mature flower buds (stage 11; **Fig. 6F, H**) than in wild-type anthers (**Fig. 6G, I**) as observed by the much lighter brown precipitate in anther tissues. A similar reduction of H_2O_2 accumulation was also observed in 35S::*ADR* anther tissue in mature flowers (stage 13; **Fig. 6J, L, M**) than in wild-type anthers (**Fig. 6K, L, N**). Pollen grains from both wild-type and 35S::*ADR* flowers showed a similar level of brown precipitate (**Fig. 6H, I, L**).

This result further supports the hypothesis that the developmental processes of anther dehiscence in 35S::*ADR* plants is interrupted by reduced ROS accumulation and the subsequent failure of lignification as well as secondary wall thickening in endothecial cells of the anthers.

External application of H_2O_2 rescued anther indehiscence in 35S::*ADR* flowers

To further investigate whether the supply of H_2O_2 affects anther indehiscence in 35S::*ADR* flowers, 1 mM H_2O_2 was externally applied to the bud clusters of the 35S::*ADR* plants. Similar to the development of wild-type flower, anther dehiscence and normal silique elongation (**Fig. 6O, P**) were observed in the 35S::*ADR* flowers 7 days after H_2O_2 treatment. Further analysis indicated that the

ovules from H_2O_2-treated 35S::*ADR* flowers were able to develop into normal embryos (**Fig. 6Q**). In contrast, silique elongation was not observed in H_2O_2-untreated mock flowers throughout the flower development (**Fig. 6O, P**). This result confirmed that the indehiscent anther phenotype in 35S::*ADR* flowers is due to the reduction of the ROS accumulation and can be complimented by exogenous H_2O_2.

Discussion

In this study, the *ADR* gene in *Arabidopsis* was functionally analyzed. Ectopic expression of *ADR* caused indehiscent anthers and resulted in a male-sterile phenotype throughout flower development. Therefore, we proposed that the *ADR* gene is functionally related to the regulatory process of anther dehiscence. This hypothesis was supported by the expression pattern of the *ADR* gene in the anthers of the stamens during flower development. In ADR::*GUS* flowers, GUS activity was strongly detected in the anthers at early developmental stages but was significantly lower in mature flowers at late developmental stages. This pattern indicates the possibility that the function of the *ADR* gene is to negatively regulate anther dehiscence during the early stages of flower development. Anther dehiscence occurred once *ADR* expression decreased after maturation. It is reasonable to conclude that the ectopic expression of *ADR* in plants extends its suppression into the late stages of flower development and causes anther indehiscence throughout flower development.

The N-terminus of ADR is presumed to be myristoylated, and this myristoylation process is critical for its membrane association[23]. It has been reported that myristoylation alone is not sufficient to anchor a protein stably to a membrane; the N-terminal basic residues contribute to protein membrane association via electrostatic interactions with acidic phospholipids[26]. The identification of several basic residues in the N-terminus and a binding site for a

peroxisomal targeting signal (PTS) in the middle of ADR supports the idea that it has a role in controlling anther dehiscence as a membrane-associated protein in the peroxisome. Clearly, this hypothesis was supported by the results of the transient expression experiment in which the ADR protein were associated with peroxisomes. These results support the assumption that myristoylation of ADR and its presence in the peroxisomes are important for its function to prevent anther dehiscence during the early stages of floral development (**Fig. 7**).

It is interesting to explore the exact role of *ADR* in the negative regulation of anther dehiscence. The indehiscent anthers observed in 35S::*ADR* flowers were similar to those of plants with double mutations in two *NAC*-like genes, *NST1* and *NST2*, which regulate secondary wall thickening in the anther endothecium[18]. Not surprisingly, no secondary thickening or lignification occurred in the anther endothecium of 35S::*ADR* plants. Furthermore, the expression of *NST1* and *NST2* was clearly down-regulated in 35S::*ADR* plants. Thus, these two genes are likely to function downstream and be negatively regulated by *ADR*.

The next question is how ADR functions and subsequently regulates secondary wall thickening in the anther endothecium. It is also known that lignin is the major compound involved in secondary wall thickening in anthers, and its polymerization is catalyzed by peroxidases that use H_2O_2 as a substrate[10-13]. Thus, ADR could possibly function to suppress lignification by decreasing H_2O_2 accumulation and disable secondary wall thickening in the endothecial cells of anthers. In this study, we found that H_2O_2 accumulation in 35S::*ADR* anther tissue is inhibited. This result strongly indicates that *ADR* suppresses anther dehiscence in 35S::*ADR* plants primarily due to the reduction in H_2O_2 accumulation and subsequent failure of both lignification and secondary wall thickening in the endothecial cells of the anther (**Fig. 7**). Following lignin polymerization, excess H_2O_2 can diffuse into the cytoplasm and regulate the expression of nuclear genes (**Fig. 7**), a process called retrograde signaling[34-36]. Thus, the reduction of H_2O_2 accumulation in 35S::*ADR* plants may suppress

NST1/2 expression in the nucleus and result in the enhancement of the anther indehiscence phenotype, as observed in our results.

Our results reveal a possible model for the interaction of *ADR* and ROS accumulation in regulating anther dehiscence in *Arabidopsis*, illustrated in **Figure 7**. In wild-type *Arabidopsis*, the high level of *ADR* expression and the target of myristoylated ADR in anther peroxisomes during early stamen development results in the subsequent suppression of H_2O_2 accumulation, *NST1/2* expression, lignification and secondary wall thickening in endothecial cells, thereby preventing early anther dehiscence. During late flower development, *ADR* expression is reduced and no longer suppresses H_2O_2 production and the processes listed above. Thus, these processes are activated in the stamen, resulting in the initiation of anther dehiscence and the release of mature pollen. In 35S::*ADR* plants, the production of H_2O_2 and subsequent processes were suppressed due to the high level of *ADR* expression at all stages of flower development. The suppression of these processes causes the subsequent failure of secondary wall thickening in the anther endothecial cells and results in anther indehiscence throughout flower development, similar to the phenotypes observed in *nst1/2* double mutations.

In conclusion, we demonstrated that myristoylated ADR associates with the peroxisome and negatively regulates anther dehiscence by promoting ROS scavenging, ultimately affecting lignin polymerization and stomium rupture in Arabidopsis.

Materials and Methods

Plant materials and growth conditions

The T-DNA insertion mutants of *ADR* (SALK_072305) *Arabidopsis* seeds were obtained from the Arabidopsis Biological Resource Center, Ohio State University, Columbus, OH, USA. *Arabidopsis* seeds were sterilized and placed on agar

plates containing 1/2 X Murashige & Skoog medium[37] at 4°C for 2 days. The seedlings were then grown in growth chambers under long-day conditions (16-h light/8-h dark) at 22°C for 10 days before being transplanted to soil. The light intensity of the growth chambers was 150 μE m^{-2} s^{-1}.

RNA isolation and RT-PCR analysis

Total RNA isolation from *Arabidopsis* plants for use in RT-PCR analysis was described previously[38]. Plant tissues were harvested, and the RNA was isolated using TRIzol Reagent (Invitrogen, USA). After being treated with DNase I (Promega), 2 μg of total RNA was subjected to reverse transcription reaction using the IMPROM II reverse transcriptase (Invitrogen, USA) at 25°C for 5 min, 42°C for 1 h and 70°C for 15 min. The resulting cDNA was used for PCR amplification with the gene-specific primers. The *Arabidopsis ACTIN8* (*ACT8*) gene was used as internal controls. Sequences of the primers used are listed in Supplementary Table S1.

Cloning of the cDNA of *ADR* from Arabidopsis

ADR (At4g13540), which contains two exons and one intron, was identified on chromosome four. cDNA containing an open reading frame of *ADR* was amplified by RT-PCR using the 5' primer ADR-cDNA-For and the 3' primer ADR-cDNA-Rev. The former contained the generated *Xba*I recognition site (5'-TCTAGA-3'), and the latter contained the generated KpnI recognition site (5'-GGTACC-3') to enable the cloning of *ADR* cDNA. To generate the *ADR-Gly* fragment, cDNA containing an open reading frame of *ADR* with the first two glycine codons removed was amplified by PCR using the 5' primer ADR-dsc-dG-For and the 3' primer ADR-cDNA-Rev. The former contained the generated *Pst*I recognition site (5'-CTGCAG-3') to enable the cloning of *ADR-Gly* cDNA. Sequences of the primers are listed in Supplementary Table S1.

The amplified fragment containing the cDNA of the *ADR* or *ADR-Gly* was

cloned into the linker region in the binary vector pEpyon-12K[39] (CHY Lab, Taichung, Taiwan) under the control of cauliflower mosaic virus (CaMV) 35S promoter (35S::*ADR* and 35S::*ADR-Gly*) and used for further plant transformation.

ADR::*GUS* fusion construct

For the ADR::*GUS* construct, the *ADR* promoter (1.6 kb) was obtained by PCR amplification from the genomic DNA using the pADR-For and pADR-Rev primers and then cloned into pGEMT easy vector (Promega, Madison, WI, USA). The full-length promoter of *ADR* (1.6 kb) was then subcloned into the linker region before the β-glucuronidase (GUS) coding region in the binary vector pEpyon-01K[39]. The primers contained the generated *Pst*I (5'-CTGCAG-3') recognition site and *Xba*I (5'-TCTAGA-3') recognition site to enable the cloning of the promoter. Sequences of the primers are listed in Supplementary Table S1.

Construction of the ADR+GFP, CAT3-mORG2 and MT-RK constructs

To generate the *ADR* fragment, cDNA containing an open reading frame of *ADR* without the stop codon was amplified by PCR using the 5' primer ADR-dsc-For and the 3' primer ADR-dsc-Rev. The amplified *ADR* fragment was then cloned into the linker region in the binary vector pEpyon-32H (CHY Lab, Taichung, Taiwan) upstream of the GFP fragment to generate the *ADR+GFP* construct, which were under the control of cauliflower mosaic virus (CaMV) 35S promoter.

To generate the *CAT3* fragment, cDNA containing an open reading frame of CAT3 without the stop codon was amplified by PCR using the 5' primer CAT3-For and the 3' primer CAT3-dsc-Rev. The amplified *CAT3* fragment was cloned into the linker region in the binary vector pEpyon-34K (CHY Lab, Taichung, Taiwan) upstream of the mORANGE2 fragment to generate the *CAT3-mORG2* construct, which was under the control of cauliflower mosaic virus (CaMV) 35S promoter. Sequences of the primers used are listed in

Supplemental Table S1. Mitochondria fusion binary plasmid (CD3-991) which contained mitochondria marker fused with mCherry fluorescent protein was obtained from the ABRC (clone name: MT-RK)[40].

Transient gene expression in leaf epidermal cells of *Nicotiana benthamiana*

Nicotiana benthamiana leaves were infiltrated with *Agrobacterium* strain C58C1 containing the pEpyon-32H vector carrying *ADR+GFP* fragment, the pEpyon-34K vector carrying *CAT3-mORG2* fragment, and MT-RFP using the methods described previously[41]. Infiltrated leaves were observed using a confocal microscope (Olympus FV1000). For ADR-GFP, excitation by 488 nm laser; and fluorescence was collected at 490-535 nm. For CAT3-mORG2, excitation by 543 nm laser, and fluorescence was collected at 550-650 nm. For MT-RK, excitation by 543 nm laser, and fluorescence was collected at 580-640 nm.

Real-time PCR analysis

For real-time quantitative PCR, the reaction was performed on an MJ Opticon system (MJ Research, Waltham, MA) using SYBR Green Real-Time PCR Master Mix (Toyobo Co., Ltd.). The amplification conditions were 95°C for 10 minutes followed by 40 cycles of amplification (95°C for 15 seconds, 58°C for 15 seconds, and 72°C for 30 seconds, followed by plate reading) and melting (50-95°C, with plate readings every 1°C). The sequences of the primers that were used for the real-time quantitative RT-PCR for *ADR*, *NST1* and *NST2* are listed in Supplementary Table S1. The Arabidopsis housekeeping gene *UBQ10* was used as a normalization control with the primers RT-UBQ10-1 and RT-UBQ10-2. All of the experiments were repeated at least twice for reproducibility. The data were analyzed using Gene Expression Macro software (version 1.1, Bio-Rad) according to the manufacturer's instructions. The 'delta-delta method' formula $2^{-[\triangle CP\ sample - \triangle CP\ control]}$, where 2 represents perfect PCR efficiency, was used to

calculate the relative expression of the genes. To calculate the statistical significance, unpaired T-tests were used.

Plant transformation and transgenic plant analysis

Constructs in this study were introduced into *Agrobacterium tumefaciens* strain GV3101 and transformed into *Arabidopsis* plants using the floral dip method as described elsewhere[42]. Transformants that survived in the medium containing kanamycin (50 μg ml^{-1}) were further verified by PCR and RT-PCR analyses.

Histochemical GUS assay

Histochemical staining was performed under the standard method described previously[43,44].

Alexander's staining

For pollen analysis, pollen grains were mounted with Alexander's stain as previously described[30].

Scanning electron microscopy (SEM)

Scanning electron microscopy was performed according to the methods described previously[45-47]. Various floral organs were fixed in 2% glutaraldehyde in 25 mM sodium phosphate buffer (pH 6.8) at 4°C overnight. After dehydration in a graded ethanol series, specimens were critical-point dried in liquid CO_2. The dried materials were mounted and coated with gold-palladium in a JBS sputter-coater (model 5150). Specimens were examined with a field emission scanning electron microscope (JEOL JSM-6700F, Japan) with an accelerating voltage of 15 kV.

Lignin staining

For lignin analysis, fresh anthers were stained with 0.01% auramine O[48] and 0.5

mg ml^{-1} Calcofluor white[49] and observed using a confocal microscope (Olympus FV1000). The lignified cells were observed under 488 nm excitation/510-560 nm emission as described previously[50].

Application of H_2O_2

For H_2O_2 complement experiment, 1 mM H_2O_2 solution containing 0.01% silwet L-77 was prepared and externally applied by dropping to the bud clusters of the 35S::*ADR* plants. The development of the silique for H_2O_2 treated flower buds was recoded and pictures were taken 10 days after treatment.

DAB staining

The H_2O_2 staining agent DAB (D5637, Sigma-Aldrich) was dissolved in water, after which the pH was adjusted to 3.8 with KOH. Inflorescences with flowers were treated with 10 μM DAB staining solution under vacuum for 4 hours. Samples were then incubated in 90% ethanol at 70°C for 10 min to remove chlorophyll. A dark brown color was visualized for H_2O_2 due to the oxidization of DAB.

References

1. Smyth, D.R., Bowman, J.L. & Meyerowitz, E.M. Early flower development in Arabidopsis. *Plant Cell* **2,** 755–767 (1990).
2. Sanders, P.M. *et al.* Anther developmental defects in Arabidopsis thaliana male-sterile mutants. *Sex Plant Reprod.* **11,** 297–322 (1999).
3. Dawson, J. *et al.* Characterization and genetic mapping of a mutation (ms35) which prevents anther dehiscence in Arabidopsis thaliana by affecting secondary wall thickening in the endothecium. *New Phytol.* **144,** 213-222 (1999).
4. Scott, J.R., Spielman, M. & Dickinson, H.G. Stamen structure and function.

Plant Cell **16,** S46–S60 (2004).

5. Wilson, Z.A., Song, J., Taylor, B. & Yang, C. The final split: The regulation of anther dehiscence. *J. Exp. Bot.* **62,** 1633–1649 (2011).
6. Cecchetti, V. *et al.* Auxin controls Arabidopsis anther dehiscence by regulating endothecium lignification and jasmonic acid biosynthesis. *Plant J.* **74,** 411–422 (2013).
7. Yang, C.Y. *et al.* Arabidopsis MYB26/MALE STERILE35 regulates secondary thickening in the endothecium and is essential for anther dehiscence. *Plant Cell* **19,** 534-548 (2007).
8. Keijzer, C. The processes of anther dehiscence and pollen dispersal. I. The opening mechanism of longitudinally dehiscing anthers. *New Phytol.* **105,** 487–489 (1987)
9. Bonner, L. & Dickinson, H. Anther dehiscence in Lycopersicon esculentum Mill. I. Structural aspects. *New Phytol.* **113,** 97–115 (1989).
10. Kawasaki, T. *et al.* Cinnamoyl-CoA reductase, a key enzyme in lignin biosynthesis, is an effector of small GTPase Rac in defense signaling in rice. *Proc. Natl. Acad. Sci. USA* **103,** 230–235 (2006).
11. Boerjan, W., Ralph, J. & Baucher, M. Lignin biosynthesis. *Annu. Rev. Plant Biol.* **54,** 519–546 (2003).
12. Ralph, J. *et al.* Lignins: natural polymers from oxidative coupling of 4-hydroxyphenylpropanoids. *Phytochem. Rev.* **3,** 29–60 (2004).
13. Yoo, K.S. *et al.* Single cystathionine b-Synthase domain–containing proteins modulate development by regulating the thioredoxin system in Arabidopsis. *Plant Cell* **23,** 3577-3594 (2011).
14. Liu, Q., Luo, L. & Zheng, L. Lignins: Biosynthesis and Biological Functions in Plants. *Int. J. Mol. Sci.* **19 (2),** 335 (2018).
15. Villarreal, F. *et al.* Ectopic expression of mitochondrial gamma carbonic anhydrase 2 causes male sterility by anther indehiscence. *Plant Mol. Biol.* **70,** 471-485 (2009).

16. Bienert, G.P., Schjoerring, J.K. & Jahn, T.P. Lignins: Membrane transport of hydrogen peroxide. *Biochimica et Biophysica Acta (BBA) – Biomembranes* **1758 (8),** 994-1003 (2006).

17. Nyathi, Y. & Baker, A. Plant peroxisomes as a source of signalling molecules. *Biochim. Biophys. Acta.* **1763 (12),** 1478-1495 (2006).

18. Mitsuda, N., Seki, M., Shinozaki, K. & Ohme-Takagi, M. The NAC transcription factors NST1 and NST2 of Arabidopsis regulate secondary wall thickenings and are required for anther dehiscence. *Plant Cell* **17,** 2993-3006 (2005).

19. Hu, J. *et al.* Plant peroxisomes: Biogenesis and function. *Plant Cell* **24,** 2279–2303 (2012).

20. Goto-Yamada, S., Mano, S. & Nishimura, M. The Role of Peroxisomes in Plant Reproductive Processes. In: Sawada H., Inoue N., Iwano M. (eds) Sexual Reproduction in Animals and Plants. Springer, Tokyo (2014).

21. Wu, J. *et al.* Spermidine oxidase-derived H_2O_2 regulates pollen plasma membrane hyperpolarization activated Ca^{2+}-permeable channels and pollen tube growth. *Plant J.* **63,** 1042–1053 (2010).

22. Li, X.R. *et al.* Arabidopsis DAYU/ABERRANT PEROXISOME MORPHOLOGY9 is a key regulator of peroxisome biogenesis and plays critical roles during pollen maturation and germination in planta. *Plant Cell* **26,** 619–635 (2014).

23. Pierre, M. *et al.* N-myristoylation regulates the SnRK1 pathway in Arabidopsis. *Plant Cell* **19,** 2804-2821 (2007).

24. Woodward, A.W. & Bartel, B. The Arabidopsis peroxisomal targeting signal type 2 receptor PEX7 is necessary for peroxisome function and dependent on PEX5. *Mol. Biol. Cell* **16 (2),** 573-583 (2005).

25. Boutin, J.A. Myristoylation. *Cell Signal* **9,** 15–35 (1997).

26. Bentham, M., Mazaleyrat, S. & Harris, M. Role of myristoylation and N-terminal basic residues in membrane association of the human

immunodeficiency virus type 1 Nef protein. *J. Gen. Virol.* **87,** 563-571 (2006).

27. Zou, J.J. *et al.* Arabidopsis CALCIUM-DEPENDENT PROTEIN KINASE8 and CATALASE3 function in abscisic acid-mediated signaling and H_2O_2 homeostasis in stomatal guard cells under drought stress. *Plant Cell* **27,** 1445–1460 (2015).
28. Wang, R. & Brattain, M.G. The maximal size of protein to diffuse through the nuclear pore is larger than 60kDa. *FEBS Lett.* **581,** 3164-3170 (2014).
29. Zha, J., Weiler, S., Oh, K.J., Wei, M.C. & Korsmeyer, S.J. Posttranslational N-Myristoylation of BID as a molecular switch for targeting mitochindria and apoptosis. *Science* **290,** 1761-1765 (2000).
30. Alexander, M.P. Differential staining of aborted and non-aborted pollen. *Stain Technol.* **44,** 117–122 (1969).
31. Costa, A. *et al.* H_2O_2 in plant peroxisomes: an in vivo analysis uncovers a Ca2+-dependent scavenging system. *Plant J.* **62,** 760-772 (2010).
32. Bindschedler, L.V. *et al.* Peroxidase-dependent apoplastic oxidative burst in Arabidopsis required for pathogen resistance. *Plant J.* **47,** 851–863 (2006).
33. Wu, A. *et al. JUNGBRUNNEN1*, a reactive oxygen species–responsive NAC transcription factor, regulates longevity in *Arabidopsis. Plant Cell* **24,** 482–506 (2012).
34. Jung, K.W. *et al.* A Cystathionine-β-Synthase Domain-Containing Protein, CBSX2, Regulates Endothecial Secondary Cell Wall Thickening in Anther Development. *Plant Cell Physiol.* **54,** 195-208 (2013).
35. Kimura, M., Yoshizumi, T., Manabe, K., Yamamoto, Y.Y. & Matsui, M. Arabidopsis transcriptional regulation by light stress via hydrogen peroxide-dependent and -independent pathways. *Genes Cells* **6,** 607-617 (2001).
36. Yabuta, Y., Maruta, T., Yoshimura, K., Ishikawa, T. & Shigeoka, S. Two distinct redox signaling pathways for cytosolic APX induction under

photooxidative stress. *Plant Cell Physiol.* **45,** 1586–1594 (2004).

37. Murashige, T. & Skoog, F. A revised medium for rapid growth and bioassays with tobacco tissue cultures. *Physiol. Plant* **15,** 473-479 (1962).

38. Chen, W.H., Li, P.F., Chen, M.K., Lee, Y.I. & Yang, C.H. *FOREVER YOUNG FLOWER* negatively regulates ethylene response DNA-binding factors (EDFs), by activating an Ethylene-Responsive Factor (ERF), to control Arabidopsis floral organ senescence and abscission. *Plant Physiol.* **168,** 1666-1683 (2015).

39. Peng, Y.J. *et al.* A RING-Type E3 ligase controls anther dehiscence by activating the jasmonate biosynthetic pathway gene *DEFECTIVE IN ANTHER DEHISCENCE1* in *Arabidopsis*. *Plant J.* **74,** 310-327 (2013).

40. Nelson, B.K., Cai, X. & Nebenführ, A. A multicolored set of in vivo organelle markers for co-localization studies in Arabidopsis and other plants. *Plant J.* **51,** 1126-1136 (2007).

41. Wydro, M., Kozubek, E. & Lehman, P. Optimization of transient *Agrobacterium*-mediated gene expression system in leaves of *Nicotioana benthamiana*. *Acta Biochimica Polonica* **53,** 289–298 (2006).

42. Clough, S.J. & Bent, A.F. Floral dip: a simplified method for Agrobacterium-mediated transformation of *Arabidopsis thaliana*. *Plant J.* **16,** 735-743 (1998).

43. Jefferson, R.A., Kavanagh, T.A. & Bevan, M. GUS fusions: β-glucuronidase as a sensitive and versatile gene fusion marker in higher plants. *EMBO J.* **6,** 3901–3907 (1987).

44. Chou, M.L., Haung, M.D. & Yang, C.H. *EMF* interact with late-flowering genes in regulating floral initiation genes during shoot development in Arabidopsis. *Plant Cell Physiol.* **42,** 499–507 (2001).

45. Hsu, H.F. & Yang, C.H. An orchid (Oncidium Gower Ramsey) AP3-like MADS gene regulates floral formation and initiation. *Plant Cell Physiol.* **43,** 1198–1209 (2002).

46. Tzeng, T.Y. & Yang, C.H. A MADS box gene from lily (Lilium longiflorum) is sufficient to generate dominant negative mutation by interacting with PISTILLATA (PI) in *Arabidopsis thaliana*. *Plant Cell Physiol.* **42,** 1156–1168 (2001).
47. Chang, Y.Y. *et al.* Characterization of the possible roles for B class MADS box genes in regulation of perianth formation in orchid. *Plant Physiol.* **152,** 837–853 (2010).
48. Pesquet, E. *et al.* Novel markers of xylogenesis in zinnia are differentially regulated by auxin and cytokinin. *Plant Physiol.* **139,** 1821-1839 (2005).
49. Herth, W. & Schnepf, E. The fluorochrome calcofluor white binds oriented to structural polysacharide fibrils. P*rotoplasma* **105,** 129–133 (1980).
50. Shih, C.F., Hsu, W.H., Peng, Y.J. & Yang, C.H. The *NAC*-like gene *ANTHER INDEHISCENCE FACTOR* acts as a repressor that controls anther dehiscence by regulating genes in jasmonate biosynthesis pathway in *Arabidopsis*. *J. Exp. Bot.* **65,** 621-639 (2014).

Acknowledgements

This work was supported by grants to C-H Y from the Ministry of Science and Technology, Taiwan, ROC, grant number: NSC97-2313-B-005-019-MY3. This work was also financially supported (in part) by the Advanced Plant Biotechnology Center from The Featured Areas Research Center Program within the framework of the Higher Education Sprout Project by the Ministry of Education (MOE) in Taiwan.

Author Contributions

C-H. Y. developed the overall strategy, designed experiments and coordinated the project. S-Y. D. performed most of the experiments and analyzed the data. W-H. H. performed Alexander's staining and lignin staining experiment. C-H. Y. prepared and revised the manuscript.

Additional Information

Supplementary information are available at online.

Competing Interests: The authors declare no competing interests.

Legends to figures

Figure 1. Analysis of *ADR* expression in different *Arabidopsis* organs and GUS staining patterns in ADR*::GUS Arabidopsis* flowers.

(A) The detection of *ADR* expression in different *Arabidopsis* organs. The mRNA levels were determined by RT-PCR. Total RNA was isolated from 1-week-old seedlings (1W), 2-week-old seedlings (2W), rosette leaves (RL), cauline leaves (CL), roots (Rt), stems (St), floral buds (FB) and siliques (Si). The *Arabidopsis ACTIN8* (*ACT8*) gene was used as internal controls. The grouping of two gels (*ADR* and *ACT8*) was cropped from two different original gels in Fig. S4.

(B) The detection of *ADR* expression in wild-type flowers at two different developmental stages (8-11, >12). The mRNA levels were determined by real-time quantitative PCR.

(C) In stage 11 of ADR*::GUS* young floral buds, GUS activity was strongly detected in the sepals (s) and anthers (an) of stamens but relatively weakly detected in the petals (p), carpels (c) and filaments (f) of stamens.

(D) Close-up of the anther (an) from (C).

(E) In stage 13 of ADR*::GUS* mature flowers, GUS was strongly detected in sepals (s), petals (p) and carpels (c). In the stamen, GUS activity was detected in the filaments (f) but was absent in the anthers (an) of stamens.

(F) Close-up of the anther (an) from (E).

Figure 2. Transient expression of ADR+GFP and CAT3+mORG2 in tobacco cells.

(A-D) Agrobacterium-mediated transient expression of ADR+GFP (A, B) and CAT3+mORG2 (C, D) in epidermal cells of *N. benthamiana*. ADR+GFP fusion protein accumulated in organelle-like structures (arrowed in A, B) that were highly similar to peroxisomes, where CAT3+mORG2 were localized (arrowed in

C, D).

(E, F) A merged fluorescence image of (A) and (C) in (E); (B) and (D) in (F) showed the co-localization of ADR+GFP and CAT3+mORG2 (green-yellow color) in peroxisomes (arrowed). (B), (D) and (F) are the close-up images from (A), (C) and (E), respectively. Scale bars: 30 μm in (A), (C) and (E) and 5 μm in (B), (D) and (F).

Figure 3. Transient expression of ADR+GFP and MT+RK in tobacco cells.

(A-D) Agrobacterium-mediated transient expression of ADR+GFP (A, B) and MT+RK (C, D) in epidermal cells of *N. benthamiana*. ADR+GFP fusion protein accumulated in organelle-like structures (white arrows in B), whereas MT+RK was localized in mitochondria (blue arrows in D).

(E, F) A merged fluorescence image of (A) and (C) in (E); (B) and (D) in (F) showing the different localization of ADR+GFP (white arrows in F) and MT+RK (blue arrows in F). (B), (D) and (F) are close-up images (boxed) from (A), (C) and (E), respectively. Scale bars: 20 μm in (A), (C) and (E) and 5 μm in (B), (D) and (F).

Figure 4. Phenotypic analysis and the detection of gene expression in Arabidopsis plants ectopically expressing *ADR*.

(A) A severe 44-day-old 35S::*ADR* plant (35S::*ADR-1*, left) was sterile and produced short siliques (arrowed), whereas wild-type plants (WT, right) produced long, well-developed siliques (arrowed).

(B) Close-up of the inflorescences from a severe 35S::*ADR* (35S::*ADR-1*) plant during the late developmental stage.

(C) Inflorescences from a severe 35S::*ADR* transgenic line (35S::*ADR-1*, middle) that showed sterility, a medium-severe 35S::*ADR* transgenic line (35S::*ADR-2*, right) that showed partial sterility and some partially elongated siliques (arrowed), and one wild-type plant (WT, left) with fully elongated siliques (arrowed).

(D) Detection of gene expression in 35S::*ADR* transgenic Arabidopsis. mRNA accumulation for *ADR* was determined by real-time quantitative PCR. Total RNA isolated from flower buds before stage 12 of one wild-type plant (WT), one severe 35S::*ADR-1* plant (*ADR-1*) and one medium-severe 35S::*ADR-2* plant (*ADR-2*) was used as templates. The transcript levels of *ADR* were determined using three replicates and were normalized against that of *UBQ10*. Gene expression levels in 35S::*ADR* plants are presented relative to that of the wild-type plants, which was set at 1. Error bars represent the standard deviation. Each experiment was repeated twice and resulted in similar results.

(E, F) Indehiscent anthers (arrowed) were observed in 35S::*ADR* plants (E) compared to wild-type plants, which showed normal anther dehiscence and pollen (arrowed) release (F).

(G) Close-up of the 35S::*ADR* indehiscent anthers (arrowed) from (E).

(H) Close-up of the wild-type dehiscent anthers (arrowed) with released pollen (po) from (F).

Figure 5. Alexander's staining and scanning electron microscopy of pollen produced in wild-type and 35S::*ADR* flowers and cross-pollination of wild-type pollen to 35S::*ADR* plants.

(A, B) Pollen grains with normal viability (arrowed, stained dark purplish red) were observed in both the wild-type (A) and severe 35S::*ADR* anthers (B).

(C, D) Close-up of the egg-shaped wild-type (C) and severe 35S::*ADR* (D) pollen grains. Colpi (arrowed) and outer exine (ex) with a typical irregular wall structure were observed on the surface of pollen.

(E) The 35S::*ADR* flower that was manually pollinated with wild-type pollen developed well-elongated siliques (arrowed), whereas short siliques (s) developed without manual pollination.

(F) Close-up of a well-elongated silique (WT x 35S::*ADR*) (left) and a short silique (right) from (E).

(G) 35S::*ADR* ovules developed into normal embryos (arrowed) after manual pollination with wild-type (WT x 35S::*ADR*) pollen grains.

Figure 6. Lignin staining of anthers, *NST1*/*NST2* expression, DAB staining in wild-type and 35S::*ADR* flowers and the phenotypic analysis of the H_2O_2-treated 35S::*ADR* flowers.

(A-D) Anthers at stage 12 were stained with auramine O and observed by confocal microscopy (488 nm excitation/510-560 nm emission). Secondary thickening is visible in the endothecium (arrow) of the anthers of wild-type plants (A, B) but is absent in 35S::*ADR* plants (C, D). Red arrowheads indicate pollen grains. (B) and (D) are close-up images of (A) and (C), respectively.

(E) Detection of *NST1*/*NST2* expression in 35S::*ADR* transgenic Arabidopsis plants. mRNA accumulation of *NST1*/*NST2* was determined by real-time quantitative PCR. Total RNA was isolated from flowers of one wild-type (WT) plant and one 35S::*ADR* plant and used as templates. The transcript levels of *NST1*/*NST2* were determined using three replicates and normalized against *UBQ10*. The *NST1*/*NST2* expression level in 35S::*ADR* plants is presented relative to wild-type plants, which was set at 1. Error bars represent the standard deviation. Each experiment was repeated twice with similar results.

(F-I) At floral stage 11, stronger DAB staining was observed in anther tissue (arrow) of wild-type flowers (G, I) than in 35S::*ADR* flowers (F, H). (H) is a close-up image for 35S::*ADR* anther whereas (I) is a close-up image for wild-type anther. Pollen (po) was stained similarly in 35S::*ADR* and wild-type flowers.

(J-L) At floral stage 13, stronger DAB staining was observed in the anther tissue (arrowed) of wild-type flowers (K, L) than in 35S::*ADR* flowers (J, L). (L) is the close-up images for 35S::*ADR* (left) and wild-type anthers (right). Pollen (po) was stained similarly in 35S::*ADR* and wild-type flowers.

(M, N) Close-up images for 35S::*ADR* (M) and wild-type anther tissue (N) from

(L). Stronger DAB staining was observed in the anther tissue (arrowed) of wild-type (N) than in 35S::*ADR* (M) flowers

(O) 7 days after 1 mM H_2O_2 treatment, the 35S::*ADR* flower developed a well-elongated silique (arrowed), whereas short siliques (s) developed without H_2O_2 treatment.

(P) Close-up of a well-elongated silique (H_2O_2 treatment; left) and a short silique (right) without H_2O_2 treatment from (O).

(Q) The 35S::*ADR* ovules developed into normal embryos (arrows) after 10 μM H_2O_2 treatment.

Figure 7. Model for the function of *ADR* in regulating anther dehiscence in *Arabidopsis*.

In wild-type *Arabidopsis*, *ADR* is highly expressed in anthers during early anther development. The ADR protein is N-myristoylated and targeted to the peroxisomal membrane to further suppress (⊣) the ROS accumulation, impedes lignification and down-regulates genes such as *NST1/2* that participate in secondary wall thickening in endothecial cells of the anthers. This causes the inhibition of anther dehiscence during early flower development. During late flower development, a significant decrease in *ADR* expression causes an increase in ROS accumulation and lignification in the anthers and the up-regulation of *NST1/NST2*, resulting in anther dehiscence in wild-type flowers. In 35S::*ADR* plants, the ROS accumulation and lignification are suppressed by the high expression of *ADR* during all stages of flower development. This suppression prevents anther dehiscence and the release of pollen throughout flower development. The gradient of *ADR* activity is illustrated by the gradual reduction in the size of the gray bar (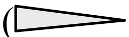) during flower maturation.
