# Supplementary material for: The Gene ANTHER DEHISCENCE REPRESSOR (ADR) Controls Male Fertility by Suppressing the ROS Accumulation and Anther Cell Wall Thickening in Arabidopsis
Source: Sci Rep. 2019 Mar 25;9:5112. doi: 10.1038/s41598-019-41382-z (PMC6434047; doi:10.1038/s41598-019-41382-z)
Supplement: Supplementary file 2 — Marked-up version of MS file [file 41598_2019_41382_MOESM2_ESM.pdf]

The Gene *ANTHER DEHISCENCE REPRESSOR* (*ADR*) Controls Male Fertility by Suppressing the ROS Accumulation and Anther Cell Wall Thickening in *Arabidopsis*

Shu-Yu Dai[1], Wei-Han Hsu[1] and Chang-Hsien Yang[1,2*]

[1]Institute of Biotechnology,
[2]Advanced Plant Biotechnology Center,
National Chung Hsing University,
Taichung, Taiwan 40227 ROC

* To whom correspondence should be addressed
E-mail: chyang@dragon.nchu.edu.tw

Dai_Fig.S1

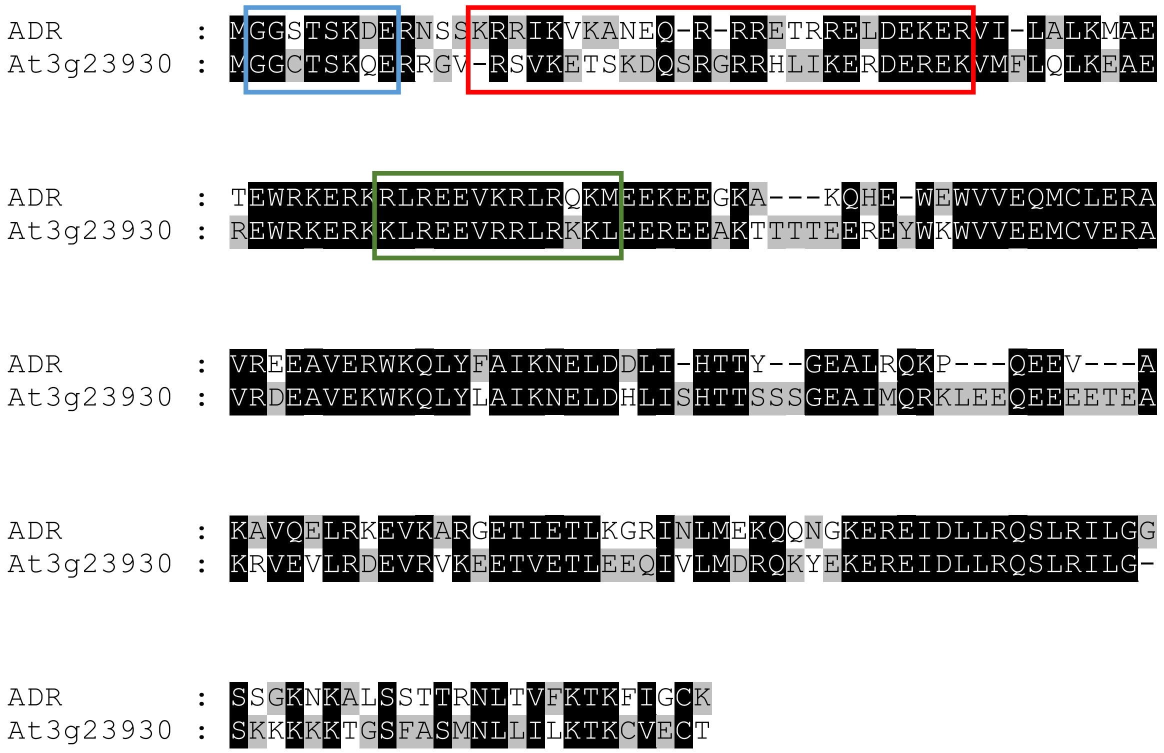

Supplementary Figure S1. Protein sequence comparison of *Arabidopsis ADR* and *ADR*-like gene At3g23930.
Protein sequence comparison of ADR (At4g13540) and other *Arabidopsis* ADR-like proteins (At3g23930). The ADR protein contains a predicted recognition site for N-myristoylase (in the blue box), N-terminal basic residues (in the red box) and a predicted binding site for a peroxisomal targeting signal (in the green box). Dashes were introduced to improve alignment. The amino acid sequences were aligned by the BIOEDIT program (http://www.mbio.ncsu.edu/BioEdit/bioedit.html) using CLUSTALW MULTIPLE ALIGNMENT with default parameters.

Dai_Fig.S2

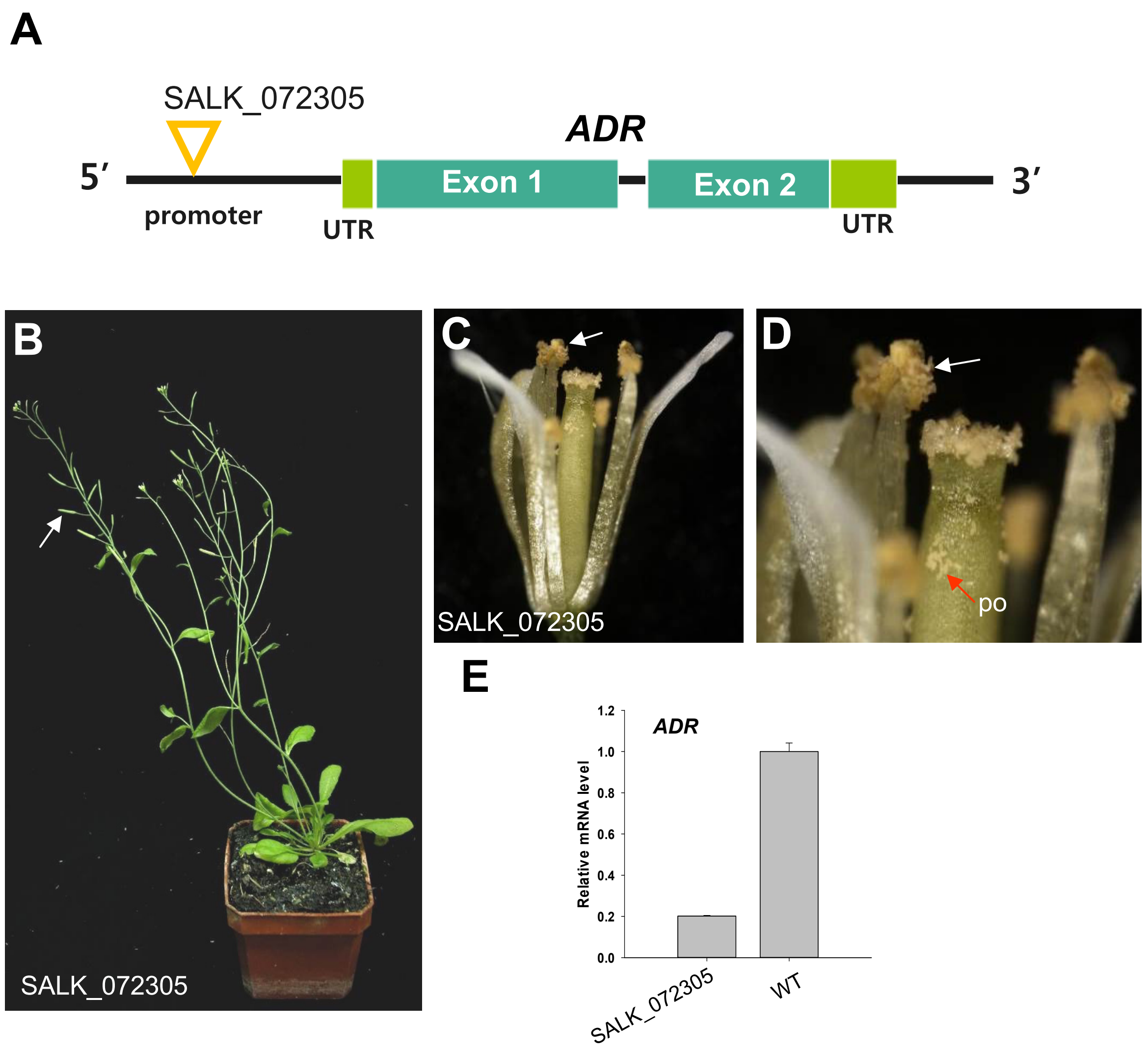

Supplementary Figure S2. Phenotypic analysis and the detection of gene expression in *ADR* T-DNA insertion line SALK_072305.

(A) Genomic region of *ADR*. Green boxes and blue boxes represent UTR and exons for *ADR* cDNA, respectively. Triangle indicates the location of T-DNA insertion in promoter region in SALK_072305 mutants.

(B) A 40-day-old SALK_072305 plant was phenotypically indistinguishable from wild-type plants and produced long, well-developed siliques (arrowed).

(C) Dehiscent anthers (arrowed) were observed in SALK_072305 flowers.

(D) Close-up of the SALK_072305 dehiscent anthers (arrowed) with released pollen (po) from **(C)**.

(E) Detection of *ADR* expression in SALK_072305 mutant and wild-type (WT) Arabidopsis. mRNA accumulation for *ADR* was determined by real-time quantitative PCR. Gene expression level in SALK_072305 plant is presented relative to that of the wild-type plant, which was set at 1.

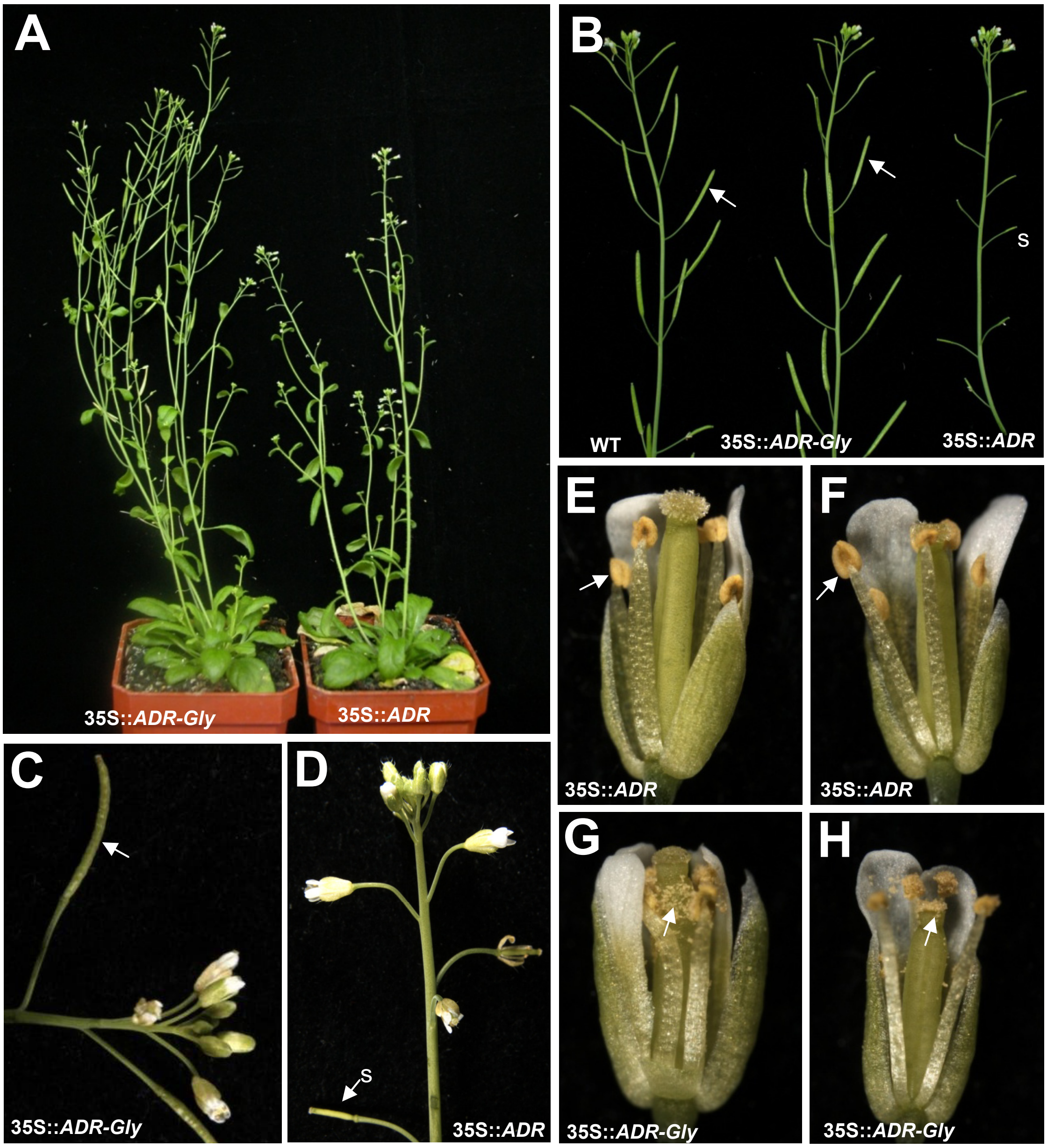

Supplementary Figure S3. Phenotypic analysis of Arabidopsis plants ectopically expressing *ADR-Gly* or *ADR*.
(A) A 46-day-old 35S::*ADR-Gly* plant (left) produced long, well-developed siliques similar to those of wild-type plants, whereas 35S::*ADR* plants produced short, undeveloped siliques (right).
(B) Inflorescences with elongated siliques (arrowed) from a wild-type plant (WT, left) and a 35S::*ADR-Gly* plant (middle), with short and undeveloped siliques (s) from a 35S::*ADR* plant (right).
(C) Close-up of a 35S::*ADR-Gly* inflorescence with flowers that developed into well-elongated siliques (arrowed).
(D) Close-up of a 35S::*ADR* inflorescence with short and undeveloped siliques (s).
(E, F) Anthers (arrowed) of 35S::*ADR* flowers remained indehiscent at stage 13 **(E)** and stage 15 **(F)**.
(G, H) Anthers of 35S::*ADR-Gly* flowers were completely dehiscent, and pollen (arrowed) was released at stage 13 **(G)** and stage 15 **(H)**.

Dai_Fig.S4

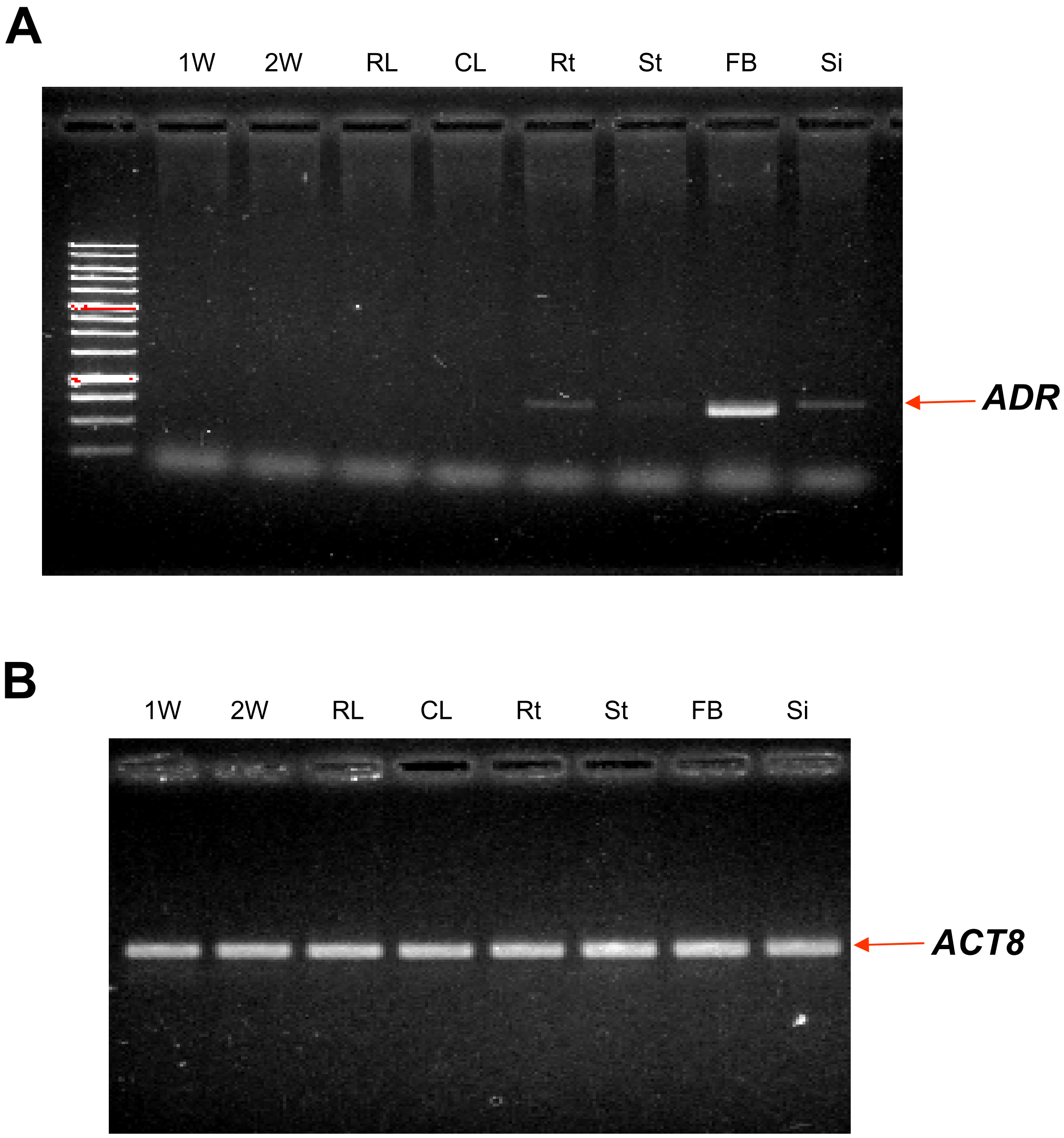

Supplementary Figure S4. Analysis of *ADR* expression in different *Arabidopsis* organs.
(A) The original gel for the detection of *ADR* expression in different *Arabidopsis* organs. The mRNA levels were determined by RT-PCR. Total RNA was isolated from 1-week-old seedlings (1W), 2-week-old seedlings (2W), rosette leaves (RL), cauline leaves (CL), roots (Rt), stems (St), floral buds (FB) and siliques (Si).
(B) The original gel for the detection of *Arabidopsis ACTIN8* (*ACT8*) gene which was used as internal controls.

Supplemental Table S1. Oligo nucleotide sequence of primers used in gene cloning and PCR analysis.

	**Primer name**	**Primer sequence**	**Restriction site**	**Use**
ADR-real-time	ADR-real-For	5’-GAGGAAAGAGAGGAAGAGACTGAG-3’		Real-time PCR
	ADR-real-Rev	5’-ACATCTGTTCCACGACCCATTC-3’		
UBQ10-real-time	RT-UBQ10-1	5’-CTCAGGCTCCGTGGTGGTATG-3’		Real-time PCR
	RT-UBQ10-2	5’-GTGATAGTTTTCCCAGTCAACGTC-3’		
ADR:GUS	pADR-For	5’-CTGCAGACCATCTGATTCTTGTATTGTC-3’	PstI	ADR Promoter cloning
	pADR-Rev	5’-TCTAGAGAAGAAAAGCTCTCTTTCTCTCTC-3’	XbaI	
35S:ADR+GFP	ADR-dsc-For.	5’-CTGCAGTCTTCATGGGTGGTTCTACAAG-3’	PstI	GFP C-ter fusion
	ADR-dsc-Rev.	5’-GGTACCTTTACACCCGATGAATTTTGT-3’	KpnI	
35S:CAT3+mORG2	CAT3-For	5'-TCAACCTTCTAGAATCACCATGGA-3'	XbaI	mORG2 C-ter fusion
	CAT3-dsc-Rev	5’-GGTACCGATGCTTGGCCTCACGT-3’	KpnI	
NST1	NST1-For	5'-GCTTAACGGACCCACATCATATTC-3'		Real-time PCR
	NST2-Rev	5'-TACGGAGATCGGACGGAAGG-3'		
NST2	NST2-For	5'-CGCCGTCGTTCAATGAGGAG-3'		Real-time PCR
	NST2-Rev	5'-TCGTGATGGTGGTGTTGTTATGG-3'		
ACT8	ACT8-For	5’-ATGAAGATTAAGGTCGTGGCACC-3’		RT-PCR
	ACT8-Rev	5’-TTATCCGAGTTTGAAGAGGCTACAA-3’		
35S::ADR	ADR-cDNA-For	5’-TCTAGAGAGAGAAAGAGAGCTTTTCTTC-3’	XbaI	cDNA cloning and RT-PCR
	ADR-cDNA-Rev	5’-GGTACCGAAGCTTACTGAGTCTACTTAT-3’	KpnI	
35S::ADR-Gly	ADR-dsc-dG-For	5’-CTGCAGCTTTTCTTCATGTCTACAAG-3’	PstI	cDNA cloning
	ADR-cDNA-Rev	5’-GGTACCGAAGCTTACTGAGTCTACTTAT-3’	KpnI	
